# Quantitative comparison of spinning disk geometries for PAINT based super-resolution microscopy

**DOI:** 10.1364/BOE.459490

**Published:** 2022-06-07

**Authors:** George Sirinakis, Edward S. Allgeyer, Jinmei Cheng, Daniel St Johnston

**Affiliations:** 1 The Gurdon Institute & the Department of Genetics, University of Cambridge, Tennis Court Road, Cambridge, CB2 1QN, UK; 2 Institute of Reproductive Medicine, School of Medicine, Nantong University, Nantong 226001, China; 3 gs519@cam.ac.uk; 4 ds139@cam.ac.uk

## Abstract

PAINT methods that use DNA- or protein- based exchangeable probes have become popular for super-resolution imaging and have been combined with spinning disk confocal microscopy for imaging thicker samples. However, the widely available spinning disks used for routine biological imaging are not optimized for PAINT-based applications and may compromise resolution and imaging speed. Here, we use *Drosophila* egg chambers in the presence of the actin-binding peptide Lifeact to study the performance of four different spinning disk geometries. We find that disk geometries with higher light collection efficiency perform better for PAINT-based super-resolution imaging due to increased photon numbers and, subsequently, detection of more blinking events.

## 1. Introduction

Single molecule localization microscopy (SMLM) is a rapidly evolving set of techniques for biological imaging applications with impressive resolution [1–3]. The underlying principle of SMLM relies on stochastically switching a sparse subset of single molecule emitters to produce a “blink” of fluorescent light, which is localized in postprocessing to determine the position of each emitter with a precision on the order of several nanometers. Widely used implementations of SMLM rely on the photophysics of fluorescent proteins and organic fluorophores to produce blinking events [4]. However, due to the complex and environmentally sensitive nature of photophysical blinking, multicolour imaging remains challenging. Blinking kinetics are also difficult to model and strongly affected by changes in excitation and activation light intensities, which complicates quantitative analysis [5,6]. Furthermore, since fluorophores are conjugated to the target proteins, photobleaching can reduce image quality and lead to inaccurate quantification. These problems are largely avoided by a different method termed points accumulation for imaging in nanoscale topography (PAINT), in which the blinking events are produced by the transient binding of exchangeable fluorescent probe [7]. A popular implementation of this approach is DNA-PAINT where a short DNA oligonucleotide (the docking strand) is attached to the protein of interest, and then imaged in a solution with freely diffusing fluorophores attached to complementary DNA oligonucleotides (the imager strands) [8]. During imaging, imager strands transiently bind to docking strands immobilizing single fluorophores to target proteins and producing blinking events. Transient protein-based interactions can also be used to target fluorescent probes to proteins of interest [9,10]. Previous work has shown that short peptides can be used to image cytoskeletal components with high labeling density [11]. PAINT-based approaches allow for more flexibility in multi-colour imaging as, apart from a larger pool of spectrally distinct fluorophores, one can multiplex by using a single fluorophore and perform sequential cycles of imaging by washing away the imager probe solution and exchange it with probes that are attached to the same fluorophore but target different proteins [12]. Binding-based kinetics are not sensitive to the properties of fluorophores or illumination parameters and can be easily tuned by adjusting the concentration of the fluorescent probes, which simplifies quantitative imaging [13]. Furthermore, PAINT is more resilient to photobleaching as fluorescent probes with bleached fluorophores are continuously replenished from the excess in the solution. However, the excess of unbound probes in solution can create large amounts of background fluorescence, compromising the ability to detect individual blinking events. Consequently, most PAINT-based applications are limited to areas close to the coverslip where selective illumination configurations such as Total Internal Reflection (TIRF) can be implemented. To overcome this problem and image deeper in cells and tissues, optical modalities that provide optical sectioning such as line scanning or spinning disk confocal have been employed [14–16]. In the latter case a rotating disk with an array of apertures is used to illuminate multiple spots in the field of view (FOV) and simultaneously collect fluorescence from the same positions [17]. The apertures are arranged in an appropriate pattern, typically along Archimedean spirals, to equally illuminate all the points in the FOV as the disk rotates at high speeds. The selection of the size, type and spacing of the apertures represents a tradeoff between the transmission efficiency of the excitation light, the amount of emitted fluorescence collected and the optical sectioning performance (background rejection) of the disk [18]. While geometries with larger and/or closer spaced apertures will increase excitation and fluorescence light throughput, they will also compromise the ability of the disk to reject out-of-focus signal and raise the level of background fluorescence due to cross talk between neighboring apertures [19]. Previous work has established best practices for several biological applications, but there is limited information for PAINT-based SMLM [20]. Here, we present experimental results on the performance of four different spinning disk geometries for PAINT-based applications. We use *Drosophila* egg chambers as a test sample and evaluate the performance of each disk geometry by repeatedly imaging the same actin structures in the presence of the actin-binding peptide Lifeact under identical conditions. We find that disk geometries with higher fill factors (aperture area/total area) tend to perform better for PAINT-based SMLM, as they allow the collection of more photons and the detection of more blinking events, which can be localized with higher precision. Sacrifices on optical sectioning appear to be less crucial but become increasingly important as the concentration of the Lifeact probes is increased.

## 2. Experimental methods

### 2.1 Optical setup

**Fig. 1.**
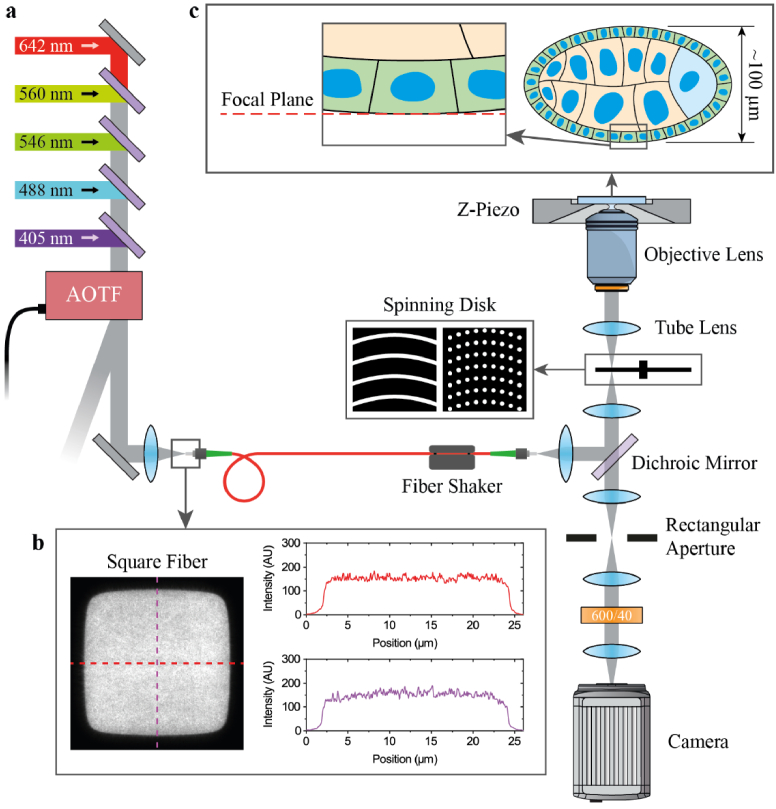
**Optical Setup. (a)** Five excitation lasers are combined, pass through an AOTF, and are coupled into a square core multimode fiber with shaker that produces uniform illumination over the field of view **(b)**. After exiting the fiber, excitation light is reflected from a dichroic mirror and illuminates a spinning disk with either a spiral or pinhole pattern. The spinning disk is imaged onto the sample plane using a tube and objective lens. **(c)** Fluorescence from basal actin of follicular epithelial cells in a *Drosophila* egg chamber is collected by the same objective and imaged onto the spinning disk. An intermediate image plane is formed at the exit of the spinning disk where a rectangular aperture is set to match the fluorescence emission area. Finally, fluorescence is relayed onto an sCMOS camera, and the resulting image is transferred to a computer.

As presented in **Fig. 1**, five lasers at 405 (Coherent, Obis 405 nm LX), 488 (Coherent, Obis 488 nm LS), 546 (MPB Communications, 2RU-VFL-P-1000-546-B1R), 560 (MPB Communications, 2RU-VFL-P-2000-560-B1R) and 642 nm (MPB Communications, 2RU-VFL-P-2000-642-B1R) are combined using dichroic mirrors and pass through an acousto-optic tunable filter (AOTF) (AA Opto-Electronics, AOTFnC-400.650-TN) for power modulation. After exiting the AOTF, the combined beams are coupled into a 150 × 150 µm square-core multimode fiber (ThorLabs, M102L05) connected to the input arm of a CrestOptics X-Light V2 spinning disk head. After the excitation laser light exits the fiber, it is reflected from a dichroic mirror (Chroma, ZT473/543/640rpc-UF1) and the fiber tip is imagined onto the spinning disk. The fiber is connected to a fiber shaker (Cairn Research, Despeckler) to homogenize the illumination. The two X-Light V2 disks have four patterns in total, either 40 or 70 µm pinholes on the first disk or 70 or 100 µm wide spirals slits on the second disk. Switching between a disk’s small or large pinhole or spirals is carried out with computer control while switching between disks (pinholes or spirals) is done manually by removing one disk and installing the other. The disk is imaged onto the sample plane using a tube lens and a 100X 1.35 NA silicone immersion objective lens (Olympus, UPLSAPO100XS) mounted in an inverted microscope base (Olympus, IX83P2ZF). During imaging, the disk is spun at 15000 RPM. Fluorescence is collected by the same objective and imaged onto the disk, passing through the aforementioned dichroic mirror, and brought to focus at an intermediate image plane with a rectangular aperture to limit the detection area. The intermediate image is relayed onto an sCMOS camera (Hamamatsu, Orca-Flash 4.0V3) while passing through a bandpass filter (Chroma, ET600/40m). Hardware control and data collection is carried out using a custom microscope control program developed in the LabView environment and freely available on GitHub (https://github.com/Gurdon-Super-Res-Lab/Microscope-Control).

### 2.2 Spinning disk geometries

**Fig. 2.**
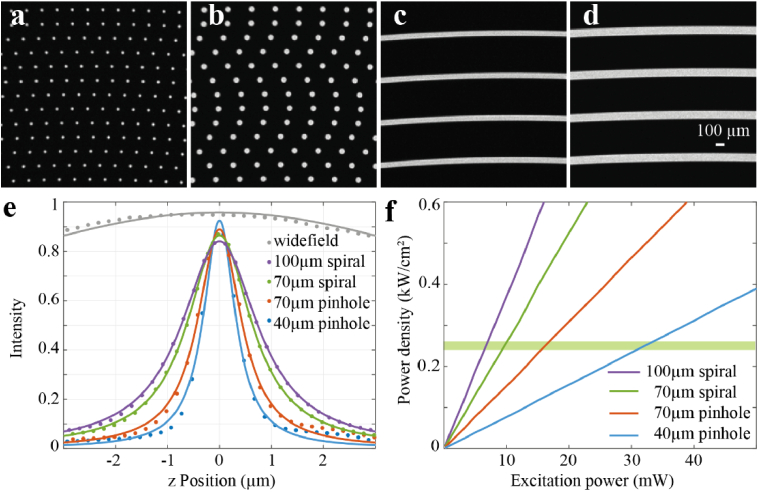
Spinning disk geometries. **(a)** 40 µm pinhole pattern with 4% fill factor. **(b)** 70 µm pinhole pattern with 8% fill factor. **(c)** 70 µm spiral pattern with 13% fill factor. **(d)** 100 µm spiral pattern with 17% fill factor. **(e)** Integrated fluorescence intensity from a bead sample as a function of axial distance from focus of the four disk patterns. The case of widefield imaging is also shown for comparison purposes. **(f)** Power density at the sample plane as a function of excitation laser power for the four different disk patterns. The power density was kept constant at 0.25 kW/cm^2^ for all disk patterns during imaging, which required 7, 9, 16 and 30 mW of laser for the 100 µm spiral, 70 µm spiral, 70 µm pinhole, 40 µm pinhole pattern, respectively, because of the different disk fill factors.

The spinning disk unit was an X-Light V2 from CrestOptics. The unit featured an exchangeable spinning disk box which was originally equipped with a standard disk with two patterns, one with 40 µm pinhole and the second with 70 µm pinholes (Fig. 2(a), b). We also used a custom disk with two patterns, one with 70 µm and a second with 100 µm continuous spirals. The centre-to-centre spacing between the spirals was 500 µm in both patterns (Fig. 2(c), d). Spirals are more effective than pinholes in increasing the fraction of the disk area that contains apertures, or the fill factor of the disk, without reducing the separation between apertures, thereby maintaining low crosstalk between apertures. Fill factors (aperture area/total area) for each disk geometry were calculated by binarizing the image of each disk pattern and then dividing the number of bright pixels by the total number of pixels in the image.

The unit was originally equipped with a 0.39 NA, 1.5 mm core multi-mode fiber that could illuminate a round field of view of ∼220 µm in diameter with the 100X objective used in this work. However, such large FOVs are impractical, as it would require several watts of lasers power to achieve power densities of 0.2∼2 kW/cm^2^, which are typically required for most PAINT-SMLM applications. To reduce the FOV we replaced the original fiber with a 0.39 NA, 150 µm x 150 µm square-core multimode fiber (Thorlabs, M102L05). In this way, we achieved uniform illumination over a 22 µm x 22 µm area without any further modifications in the illumination optics of the spinning disk unit and reached the excitation intensities used in this work with less than 50 mW of laser power. This makes PAINT based approaches possible without the use of a micro-lens array and relatively modest laser powers. Additionally, the X-Light system uses standard sized dichroic mirrors and filters and may be easily modified onsite.

### 2.2 Drift correction

Unlike cultured cells, *Drosophila* egg chambers do not tightly adhere to slides or coverslips, making real-time axial drift correction necessary for SMLM image acquisition. We therefore integrated an additional beam path into the optical setup as previously described by Cheng *et al.* [15] and following from the work of McGorty and co-workers [21]. In brief, a near-IR (NIR) light emitting diode lamp was added to the transmitted light illumination column of the inverted microscope base. This allowed Köhler illumination in the NIR range, outside the fluorescence emission and collection band. An additional dichroic mirror was added just below the objective lens to reflect NIR light out of the microscope, through a lens, and onto an inexpensive camera. This allows transmitted light images to be collected simultaneously with SMLM data acquisition. Prior to data collection, a z-stack of reference transmitted light images is collected. During SMLM data acquisition, transmitted light images are collected and cross-correlated with the reference images to determine the axial and lateral drift. Axial drift is corrected on the fly while the lateral drift is corrected after each block of 1000 frames. The localized data is further corrected for lateral drift in post-processing using the redundant cross correlation correction algorithm from Wang and coworkers [22].

### 2.3 Data acquisition and analysis

SMLM data was acquired with the microscope as described above. Raw camera frames were acquired in blocks (cycles) of 1000 at 10 frames per second (FPS). In total, 10,000 frames were collected for each image. Between the collection of each 1000 frame cycle, the lateral sample drift was corrected, if necessary, as described above. The drift correction method described above allows the (x, y, z) sample position to be maintained during imaging and while switching between different disk geometries. To reduce egg chamber to egg chamber variability, each egg chamber was imaged with all four spinning disk patterns and with widefield (no disk). The disk patterns and widefield were also randomly permuted to avoid bias towards a specific disk pattern. As mentioned above, while switching between patterns, the sample position was maintained using our drift correction method ensuring that the same FOV was imaged for each pattern. The average laser power at the sample was kept constant as 0.25 kW/cm^2^ across all disk geometries. Single-molecule events detected in raw camera frames were fit with a 2D Gaussian model using the fit package available from Li *et al.* [23]. Fits were filtered based on number of photons (50 to 5000), localization precision (0.5 to 30 nm), PSF width (50 to 150 nm), and log-likelihood ratio. Images were reconstructed from fitted positions with a 10 nm pixel size.

### 2.4 Optical sectioning performance

Optical sectioning measurements were performed with an Olympus 100X, 1.35 NA silicon oil objective and a single layer of 100 nm Crimson (Invitrogen, C47248) beads immobilized on a coverslip. The beads were mounted in Olympus silicon oil and scanned along the Z direction with a step size of 200 nm under 642 nm excitation, collecting 10 frames through a Chroma bandpass filter (ET705/72) at each Z position. Intensity values at each position were obtained by first calculating a 10-frame average for the intensity of each pixel followed by an averaging over all the pixels in the FOV. Subsequently the intensity values at each Z position were normalized to the maximum value, plotted as a function of Z position, and fitted with a mixture of a Lorentzian function for full width at half maximum (FWHM) calculation and a gaussian function for background subtraction.

### 2.5 Sample preparation

*Drosophila* egg chambers were prepared as previously described with minor modifications [15]. Briefly, *w*^1118^ flies were fattened on yeast at 25°C for one to two days before dissection. Their ovaries were dissected in Schneider’s medium (Merk, S0146) at room temperature and the muscle sheath surrounding the egg chambers was removed. Egg chambers were put into warm (38°C) fixation buffer (4% methanol-free formaldehyde (16% (w/v) Paraformaldehyde, Fisher Scientific, 28908), 2% Tween 20 (Merk, P1379) in 0.5x Phosphate buffered saline (PBS)) for 20 minutes at room temperature with rotation and then washed 3 × 10 minutes with 0.2% Tween 20 in PBS. Individual egg chambers were attached to the coverslip bottom of an 8-well chambered slide (Ibidi, µ-slides 80827) with Cell-Tak (Corning, 354240), and mounted in 300 µl of imaging solution. The final dimensions of the volume occupied by the sample and the imaging solution were 9.4 mm x 10.7 mm x 1.0 mm (width x length x height). The imaging solution consists of 2 nM Cy3B Lifeact and a high ionic strength PBS-based buffer as 1X PBS, 500 mM NaCl (Merk, S7653), 20 mM Na2SO3 (Merk, S0505) and 1 mM (±)-6-Hydroxy-2,5,7,8-tetra methylchromane-2-carboxylic acid (Trolox, Merk, 238813-1G), with a pH between 7.3-7.5. N-terminally labelled Cy3B Lifeact was synthesised by Peptide Protein Research Ltd. Lifeact Cy3B was stored at -20°C at a concentration of 1 µM stock in 50 mM KCl, 1 mM EDTA, 100 mM Tris–HCl, pH 8.0 and 50% glycerol, and serially diluted into imaging buffer. Trolox was dissolved directly in methanol to give a 100 mM solution (following from Cordes *et al.* [24]) and was diluted before use to 1 mM in the imaging buffer. It was stored at -20°C.

## 3. Results and discussion

To evaluate the performance of the 4 different disk patterns for PAINT-based SMLM we first measured the optical sectioning of each pattern using 100 nm crimson beads. As expected, the 40 µm pinhole had the best optical sectioning with a FWHM of 0.738 µm followed by the 70 µm pinhole pattern with 1.011 µm. The 70 and 100 µm spirals exhibited worse optical sectioning with 1.497 µm and 1.789 µm FWHMs respectively, but were more efficient in excitation light transmission Fig. 2. To facilitate the comparison between the different disk patterns we maintained a constant excitation power density at the sample plane of 0.25 kW/cm^2^ during imaging, which minimized photobleaching of the Lifeact probes, while still providing sufficient numbers of photons per binding events for all the disk geometries. This value could be obtained with 7 mW of 546 nm laser power for the 100 µm spirals (lowest), but it required 30 mW (highest), to achieve the same level of power density with the 40 µm pinholes (Fig. 2(c)).

Next, we investigated how the different disk patterns affect the number of photons, background, and localization precision per blinking event, as well as the number of detected blinks. To facilitate a side-by-side comparison and minimize sample-dependent effects, we imaged the same basal actin structures in stage 6 or 7 *Drosophila* egg chambers with each disk pattern. We also collected one additional dataset for each actin structure in the absence of a spinning disk under widefield illumination, to provide more context for the comparison. Switching between patterns located on the same disk was achieved using the software interface while switching between the physical spinning disks required manually removing one disk unit and replacing it with the other. While switching between the different spinning disk units or patterns, the real-time drift correction described above was continuously running to maintain the sample position. For each pattern we recorded 10000 frames at a rate of 10 Hz. To evaluate how the background fluorescence levels affect the performance of the different patterns we worked with a “low” and “high” concentration of Lifeact probes at 2 nM and 4 nM respectively. Higher concentrations were not investigated to avoid artifacts from overlapping blinks.

**Fig. 3.**
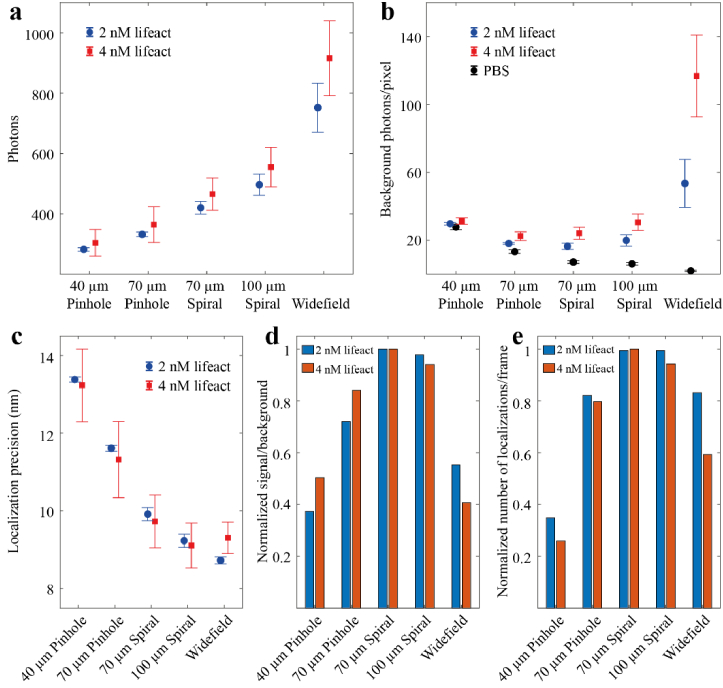
Effect of disk geometry on localization parameters. **(a)** Mean number of photons per localization. **(b)** Mean number of background photons/pixel. **(c)** Mean localization precision. Data are plotted for each one of the four disk patterns at 2nM (blue circles) and 4 nM (red squares) Lifeact concentration. Error bars indicate mean ± s.d. (n = 3 for 2 nM Lifeact and n = 4 for 4 nM Lifeact). Dark circles in **(b)** indicate background photons/pixel when imaging in PBS buffer in the absence of any fluorophore. **(d)** Normalized signal/background ratio for each disk pattern at 2 and 4 nM Lifeact concentration. **(e)** Normalized number of localizations/frame for each disk pattern at 2 and 4 nM Lifeact concentration

As expected, the disk pattern with 40 µm pinholes, which for our experimental conditions corresponds to 0.74 Airy units, had the worst performance, and exhibited lower photon numbers (295 photons) than the pattern with the 70 µm pinholes (1.3 Airy units, 350 photons) as the larger pinhole size allows for more efficient collection of the light from in-focus blinks. Photon numbers further increased to 446 for the 70 µm spirals with a larger aperture than the pinhole disk pattern of the same size. Finally, the 100 µm spiral disk pattern had the highest photon numbers at 530 as its spiral size is further increased to 1.85 Airy units and featured the largest aperture of all the patterns. Under our experimental conditions, where the excitation power density is kept constant for each disk geometry, aperture sizes larger than ∼2 Airy units will have minimal impact on fluorescence light collection of in-focus emitters and photons values are expected to plateau at the levels of widefield imaging with ∼845 photons (Fig. 3(a)) [25].

The use of a spinning disk produced a significant, up to 5-fold, reduction in background levels compared to widefield imaging Fig. 3(b). Background photon numbers for each disk pattern increased with increasing Lifeact concentration. The 100 µm spiral had the highest background levels with 31 photons of background per pixel at 4 nM Lifeact, compared to the 70 µm spirals with 20 background photons per pixel at 4 nM. The number of background photons is determined from blink fitting. Surprisingly, the pinhole patterns which exhibit higher optical sectioning than the spiral patterns did not show a significant improvement in background rejection. In fact, the 40 µm pinhole pattern which features the highest optical sectioning ability had worse performance than the pattern with the 70 µm pinholes. We hypothesized that there was another source of background related to the scattering of excitation laser light from the disk itself. To test this, we measured the background generated by each disk pattern under the experimental conditions used for SMLM imaging, but using a PBS solution with no fluorescent sample present Fig. 3(b). In this case, the 40 µm pinhole pattern which also had the lowest fill factor had the highest background with 28 photons/pixel. Background is further reduced to 13 photons/pixel for the 70 µm pinhole pattern and appears to level off for the spiral disk patterns with 7 and 6 photons/pixel for the 70 µm and 100 µm spirals, respectively. In the absence of a spinning disk, we measured 2 photons/pixel of background. A major challenge for a spinning disk unit not equipped with a microlens array, is the inefficient use of the excitation light as a significant fraction is not transmitted by the disk and may be scattered backwards into the fluorescence detection path. The amount of scattered light is determined by the disk fill factor, which in our case varies from ∼4% for the 40 µm pinholes to ∼16% for the 100 µm spirals. Furthermore, under our experimental conditions, where we maintained constant power density at the sample plane, higher laser powers were required for patterns with lower fill factors. This explains the increased levels of scattered laser light for the 40 µm and 70 µm pinhole disk patterns.

The localization precision achieved with each disk pattern does not seem to be significantly affected by increasing the Lifeact concentration from 2 to 4 nM, Fig. 3(c). The 40 µm pinhole pattern had the worst performance with a localization precision of 13.3 nm, followed by the 70 µm pinhole pattern at 11.4 nm. The spiral disk patterns performed better, with a localization precision of 9.7 nm and 9.1 nm for the 70 and 100 µm spiral patterns, respectively. Widefield imaging resulted in the lowest localization precision of 8.7 nm for the 2 nM Lifeact concentration which increased to 9.3 nm for 4 nM concentration where background levels increased to 117 photons/pixel. As expected, the localization precision had an inverse relationship with the number of photons but was not strongly affected by these background levels [26]. Even when imaging under widefield conditions and increasing the Lifeact concentration from 2 to 4 nM, which doubled the background, the localization precision only increased by 7%. Taken together, these results suggest that disk patterns with higher light collection efficiency provide higher localization precision for PAINT-based applications with probe concentrations up to 4 nM. In our hands, higher probe concentrations were not practical and lead to artifacts from overlapping blinks.

To further evaluate the performance of the different disk patterns, we calculated the signal to background ratio (SBR) for each disk pattern by dividing the average number of photons by the corresponding background per pixel. Both the number of photons and background are determined from fitting single molecule blinking events during post processing. To facilitate comparison between the different disk patterns at different Lifeact concentrations, the SBRs were normalized to the maximum and plotted as a bar chart for each pattern in Fig. 3(d). For both Lifeact concentrations, the SBR increased with increasing pinhole size for the pinhole disks patterns and reached its maximum for the 70 µm spiral pattern. Increasing the spiral width to 100 µm resulted in the SBR decreasing which was further reduced for the widefield case. As the aperture size on the disk increases, both the number of photons and the background will increase. However, as the aperture size is increased above ∼1 Airy unit the rate of increase in the number of signal photons collected from in-focus blinks is expected to reduce and then plateau around ∼ 2 Airy units. In contrast, the background photons from the freely diffusing probes will continue increasing with increasing aperture size leading to a decrease in the SBR ratio [25]. The optimum aperture size will depend on the experimental conditions. Larger aperture sizes are better suited for low background levels, as shown by the higher SBR at 2 nM compared to 4 nM for the 100 µm spirals . In contrast, smaller apertures are a better choice for higher background levels, such as the pinhole patterns which have better optical sectioning and exhibit higher SBR at 4 vs 2 nM Lifeact concentration. Although these trends do not have a significant effect on the localization precision, which, under our experimental conditions, is dominated by the numbers of signal photons, SBR will affect the number of blinks that can be detected. Disks with suboptimal feature sizes will result in the rejection of more single molecule blinks as dim blinks will either be diminished in intensity by small aperture sizes or get buried in higher background levels allowed by larger apertures.

To investigate this further we calculated the average number of blinks per frame by counting the number of blinks in each frame and then averaging over the 10000 recorded frames for each disk pattern, Fig. 3(e). These numbers strongly depend on the density of actin filaments present. To minimize these sample-dependent variations we organized data collection in datasets. In each dataset the same region of an egg chamber was imaged with each disk pattern under otherwise identical conditions. However, repeat measurements were performed on different egg chambers in regions with different densities of actin filaments. To compare those datasets, we normalized the data from each disk pattern within a dataset, against the disk pattern with the maximum average number of blinks per frame (Fig. 3(e)). At 2 nM Lifeact concentration, the patterns with the spirals had very similar performance and achieved the highest number of detected blinks followed by the 70 µm pinhole pattern with a 20% drop in the number of detected blinks. The 40 µm pinhole pattern had the worst performance with a 65% reduction in the number of detected blinks. However, when the Lifeact concentration increased to 4 nM, the performance of the 100 µm spiral pattern deteriorated and showed a 10% reduction in the number of detected blinks compared to the 70 µm spirals, which performed best. Widefield imaging also exhibited inferior performance relative to the 70 µm spiral pattern and had a stronger dependence on the Lifeact concentration with a 20% reduction at 2 nM Lifeact and 40% reduction at 4 nM Lifeact.

**Fig. 4.**
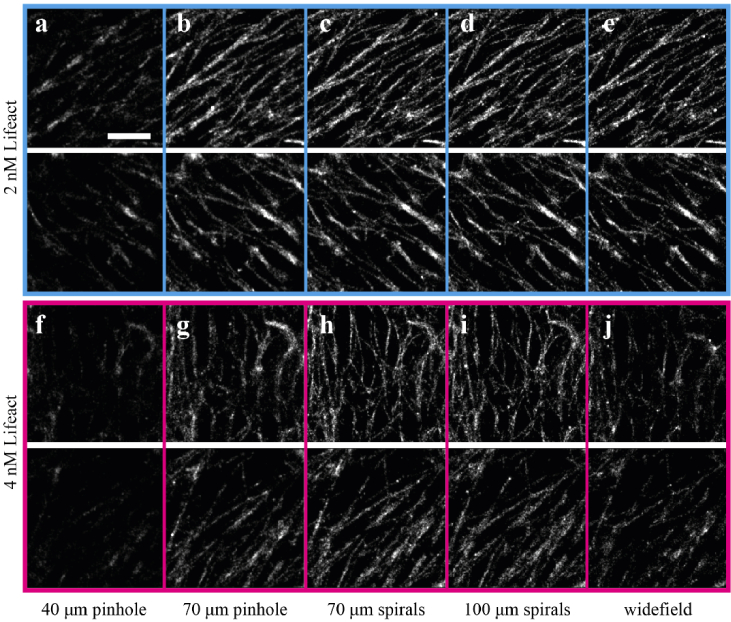
Effect of disk geometry on image quality. **(a-e)** SMLM images of basal actin in the same region of the follicular epithelium imaged with the 4 different disk patterns and widefield illumination at 2 nM Lifeact concentration. Greyscale range is kept constant for all images to facilitate comparison. **(f-g)** SMLM images of the same region imaged with the 4 different disk patterns and widefield illumination at 4 nM Lifeact concentration. Greyscale range is kept constant for all images to facilitate comparison. Scale bar 1 µm.

The reduced number of detected blinks, in certain cases, had an appreciable impact on the reconstructed images with images from the 40 µm pinhole disk pattern affected the most. This small pinhole size, due to its reduced light-collection, allowed only the brightest fraction of the in-focus blinks to be detected. Subsequently, the low photon numbers combined with the increase of background photons due to scattered laser light, resulted in poorly localized blinks and produced visually inferior reconstructed images Fig. 4(a). Increasing the aperture size to 70 µm pinholes (Fig. 4(b)) and again to 70 µm spirals (Fig. 4(c)) allowed the detection of more blinks with higher numbers of photons and produced reconstructed images where actin filaments appeared more uniformly labelled and finer details of the underlying actin structure could be resolved. Further increasing the aperture size to a 100 µm spirals did not have any significant benefit in the reconstructed image quality Fig. 4(d). Finally, under widefield imaging actin filaments were still visible but appeared more sparsely labelled, due to the lack of any optical sectioning and a reduced number of detected blinks Fig. 4(e). When the Lifeact concentration was increased to 4 nM, the 70 µm spiral pattern (Fig. 4(h)) produced images with higher quality than the 40 µm (Fig. 4(f)) and 70 µm (Fig. 4(g)) pinhole patterns and the 100 µm spirals. In the case of the 100 µm spirals, the lower optical sectioning produced higher background levels that reduced the number of detected blinks, resulting in actin filaments that appear to be more sparsely labelled Fig. 4(i). For widefield, the reduction in detected blinks was even more pronounced. The lack of optical sectioning resulted in images with inferior quality where the actin filaments appeared to be sparsely labelled and the fine details of the actin structure could no longer be resolved Fig. 4(j).

## 4. Conclusions

PAINT-based SMLM approaches which rely on the binding kinetics of freely diffusing and exchangeable probes provide a straightforward path to multicolour and quantitative imaging. However, these approaches suffer from high levels of background produced by the freely diffusing probes and their application is generally limited to cases where TIRF illumination can be employed to confine excitation to areas close to coverslip. Recently, spinning disk confocal microscopy, an established and widely used technique, was successfully used to extend the application range of PAINT-based SMLM to thicker samples further away from the coverslip. Here we show that spinning disk geometries with higher light collection efficiencies perform better for SMLM applications than the most common geometries used for standard biological imaging, with pinholes of ∼1 Airy unit. Although background suppression is important, the stochastic nature of the sparse blinking events that are imaged in SMLM allows the use of geometries with higher photon collection efficiencies and worse optical sectioning. However, a balance between background suppression and light collection needs to be found that maximizes the number of detected blinking events. Under our experimental conditions, the disk geometry with the 70 µm (1.3 Airy units) spirals had the best overall performance of the 4 patterns studied here.

## Data Availability

Data underlying the results presented in this paper are not publicly available at this time but may be obtained from the authors upon reasonable request.
